# Processing Fluorescence Spectra for Pollutants Detection Systems in Inland Waters

**DOI:** 10.3390/s20113102

**Published:** 2020-05-30

**Authors:** F. Jose Arques-Orobon, Francisco Prieto-Castrillo, Neftali Nuñez, Vicente Gonzalez-Posadas

**Affiliations:** 1 Escuela Técnica superior de Ingeniería y Sistemas de Telecomunicación, Universidad Politécnica de Madrid, 28031 Madrid, Spain; neftali.nunez@upm.es (N.N.); vicente.gonzalez@upm.es (V.G.-P.); 2 Complex Systems Group, Universidad Politécnica de Madrid, 28040 Madrid, Spain; francisco.prieto@upm.es; 3 Media Laboratory, Massachusetts Institute of Technology, Cambridge, MA 02139, USA; 4 Instituto de Energía Solar, Universidad Politécnica de Madrid, 28040 Madrid, Spain

**Keywords:** sensors, fluorescence, fluoro-sensing, spectroscopy, water pollutants

## Abstract

Development of contaminant detection systems in various natural and industrial environments has been favored in recent years thanks to the evolution of processors and sensors. Our group works specifically on contaminant detection systems in inland waters: immediate and continuous detection is a fundamental requirement in this type of sensing. Regarding the sensors, the proposed system is based on fluorescence, since it offers a method in which there is no contact with water, which means less wear on the components and a great saving in cleaning and maintenance. On the other hand, the spectrum processing is of great importance, since it is used both in the generation of a library of fluorescence spectra taken in the laboratory and in the continuous analysis of the samples and in the comparison algorithm for identification. The validity of the system is based on the last process that is carried out in a very short time. This article describes a system to process spectra in a more accelerated way.

## 1. Introduction

Fluorescence-based methods for compound detection have been used mainly in the laboratory for several years thanks to technological developments in both light emission and detection. The evolution of HP (high power) UV-LEDs (ultraviolet-light emitting diodes) at increasingly shorter wavelengths, as well as new small spectrometers, facilitate the development of lightweight contaminant detection systems for inland waters [1].

The demand for these systems has increased over time, as the implementation of water quality control stations is increasingly widespread. These systems must meet various conditions such as robustness, continuous operation, and small size. Against these advantages, the measurements are less precise than those carried out in the laboratory, but in the case explained here (detection), the speed in the treatment of the data obtained is important, since it is intended to alert about the presence of elements foreign to the aquatic environment.

The analyzers used in these stations should normally be submerged into the water, so periodic maintenance is necessary. On the other hand, fluorescence systems do not have direct contact with water, and hence, their maintenance is lower. The analyzers of basic parameters (oxygen, pH, temperature), have evolved a lot in recent years [2,3,4,5,6,7], and have been extended to other types of measurements (ammonia, carbon, phosphates, etc.) whose values must be kept within margins that depend—among others—on calendar date, environment and provenance of the water under study. Unlike these, there are substances that seriously damage the quality of water and should not be present [1,8,9,10,11].

In this paper, a method of processing the spectra obtained through a system developed by the authors is presented. It meets the aforementioned requirements since with the use of very few variables, very fast results can be obtained for the detection of contaminating substances.

Hydrocarbons have been chosen for this study since they are a very good starting point from which development can be extended [12,13]. They are on the surface, as they are less dense than water. They are also volatile and therefore they have good fluorescence. Finally, its presence is very negative for the flora and fauna of rivers and reservoirs.

## 2. Materials and Methods

To implement the processing system, it is first necessary to store substance data in a database on which the comparisons will be carried out. By using a fast processing architecture this database can then be enlarged and managed by the addition of new entries.

### 2.1. Obtaining Patterns for the Database Building

In the laboratory, the compounds were characterized by obtaining the fluorescence spectrum in a workbench with an HP-UV LED light source that emitted at an ultraviolet wavelength (365 nm) and with a power of 3 W. All measurements were made with the contaminant in distilled water at 25 °C and at fixed distances from both the emitter and the detector [1].

In this way, various hydrocarbons in common use in the automotive sector were taken by way of example to verify that even though they are similar compounds, their detection was possible by means of the fluorescence spectrum that was emitted when illuminated by an ultraviolet light source:

- Diesel A: It is used by all types of vehicles, from small size to large tonnage. Type A diesel is the most refined, producing a quality superior to the rest and making it more suitable for vehicles. Although it is thought that its use will decrease with the passage of time, it is still largely present.
- Diesel A +: The name of this type of fuel differs depending on the brand, but it is in widespread use almost everywhere in the world. This is diesel A but with added additives to improve the care and performance of the engine, since it differs in the cetane level (“ignition interval”), being in this case somewhat higher.
- Diesel B: It is the fuel for agricultural and industrial use. It is a less sensitive type of motor fuel than those that use type A. Diesel B is subsidized in many states, which offers a reduction on the taxes that are applied to it. Its use for heating is rather reduced, since its calorific value is lower than C since it contains fewer paraffins.
- Diesel C: It is the fuel used in the heating. It has a high content of paraffin and a higher density, with great calorific value. It is the one that contains the most impurities.
- Gas 95: This motor vehicle fuel is in widespread use, and therefore there is a lot of traffic that transports it in very variable amounts.
- Gas 98: Like the previous one, but it differs mainly in the octane number.

Furthermore, in order to be identified, the dyes carried by each of these fuels must be taken into account, which may influence their fluorometric response. All the compounds studied are transported and stored extensively and in different quantities ranging from tens of liters to tons, so there is a high probability of accidental spills. 

The sample was irradiated by the light source so that the compound emitted a fluorescence spectrum that was collected with a system consisting of (1) an optical fiber without collimator, and (2) a mini-spectrometer connected to a PC running MATLAB® (MathWorks®, Natick, MA, USA) program, a common tool for this objective [10,14,15,16,17], which was responsible for the processing and preliminary storage. The optical power of the spectrum was proportional to the amount of the substance present, and the evolution of HP-LEDs caused devices with more optical power to appear on the market, making calibration necessary. Despite the fact that the conditions in which the sample was found were those of the laboratory, the spectra obtained were not exempt from noise. Therefore, in a very short time interval (10 ms) five samples were taken, from which we then obtained the average. A similar process will be performed in the detection system.

To ensure that the pattern presented an allowable margin of error, various measures were taken in different procedures. This way, several steps were necessary in order to minimize the possible error in the comparison. Likewise, in the comparison process it must be taken into account that the greater the number of spectra stored, the longer the identification will take. Therefore, the development of an optimal algorithm, as well as a pattern storage system containing the minimum data possible was essential for the validity of the method.

The complete process of obtaining patterns involved the following steps:

- Measurements and averaging.
- Normalization and band limitation.
- Smoothing
- Decomposition into simple functions.
- Obtaining the relevant pattern and estimation of the error.

#### 2.1.1. Measurements and Averaging

**Figure 1:**
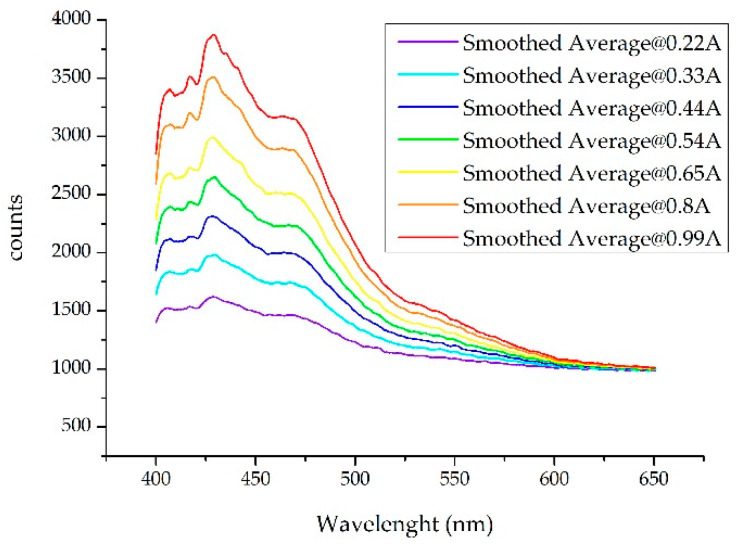
Spectra obtained when a Gas 95 sample is illuminated with an HP-UV LED (High Power Ultra-Violet Light Emitting Diode) at different working currents. The spectra shown were smoothed with the SG filter and had a greater range of wavelengths than that used for the job so that differences were better observed.

Data collection, and in particular those carried out during short exposure times, was susceptible to error. In the case at hand, where one tries to generate a warning (alarm), it is then necessary to minimize the error. With the used spectrometers (with CCD) the measurement is clearer if the exposure time is increased [1]. For example, for the detection of gasoline 95 pollutants with an optical excitation power corresponding to the nominal current (650 mA), a maximum of 2900 counts (approximately) is obtained with an exposure time of 10 ms, whereas if the exposure is 20 ms the maximum increases to 4800 counts. Therefore, it was necessary to set a trade-off so that the reading was validated while at the same time the process was fast enough. In our case, in view of the results, a measurement of 10 ms was enough. Figure 1 shows the proportional difference in optical power (in counts) of the spectrum when the compound was illuminated with the HP-UV LED at different currents. The spectra shown were smoothed with a Savitzky-Golay filter and had a greater range of wavelengths than that used for this work so that differences were better observed. It should be noted that once these responses were normalized, they were very similar, as seen below.

**Figure 2:**
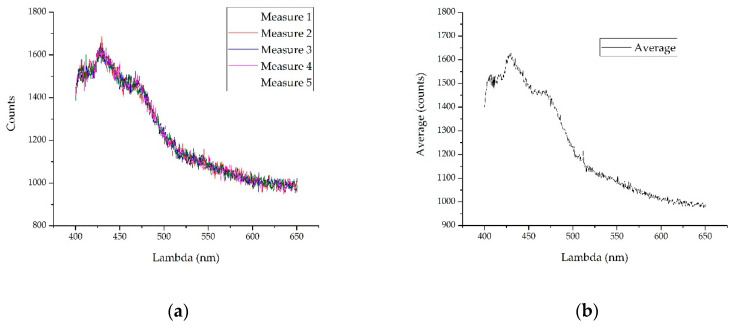
To illustrate noise reduction using the average of five measurements, the measurements made with the lowest current are shown, since proportionally the noise will be greater: (**a**) The five overlapping measurements are shown. Note a floor of approximately 1000 counts; (**b**) performing the average in the five samples, the noise obtained was lower than in any of the measures.

On the other hand, and to carry out a first noise reduction, five measures have been taken to compute the average value. Then, an average spectrum can be obtained approximately every 50 ms. The spectrometers used in the experiments shown were not without noise. It was also necessary to take into account that although the system to be developed will encounter a controlled flow of water, it was possible that there will be alterations in it, so taking several measures was necessary. Figure 2 shows an example of noise reduction when five measurements were made. In order to validate the method, the test is shown with the spectra where the signal-to-noise ratio was lower (with the spectrum with the lowest optical power obtained with the 0.22 Ampere current) so that the noise was proportionally higher. The comparison process lag described below has to be added to this step so that the whole detection analysis time can be calculated. In any case, it must result in a reduction time which is suitable for its installation in a real-time measurement system.

#### 2.1.2. Normalized and Limitation

With the purpose to obtain a robust pattern that was as independent as possible of the optical power of the reading process, it was necessary to normalize the average obtained in the previous section. Similarly, depending on the spectrometer used, spectra may appear with a ground level that does not provide information, so once the spectrum limitation has been carried out, it is necessary to eliminate this floor and then normalize. In Figure 2 it is shown that within the range provided, a subtraction of about 1000 counts can be made on the vertical axis.

In addition, in view of the results obtained with the hydrocarbons studied, the wavelengths can be considerably limited with regard to those measured by the spectrometer because information is restricted to a range narrow enough. In this way, the processing was also reduced and it can be carried out in less time.

#### 2.1.3. Smoothing

In order to discover extrema reliably, we first implemented signal smoothing (i.e., noise reduction). There are several smoothing methods that work differently depending on the nature of the signal and the noise it contains. Each type of filtering enhances or attenuates different aspects with more or less emphasis. Among the various filters that can be used to eliminate noise, three basic ones were suitable for filtering the responses obtained from the spectrometer:

- MF: Moving average (median) window filtering
- FFT: Fast Fourier Transform filtering
- SG: Savitzky-Golay filtering.

**Figure 3:**
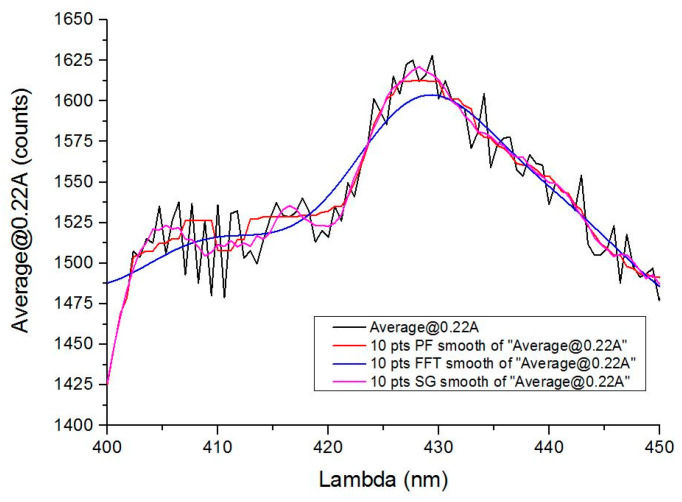
Comparison of the three types of filtering carried out with all the samples: the figure shows a detail of the measurement at 220 mA with a sample of gasoline 95. MF: median filtering. FFT: fast fourier transform. SG: Savitzky-Golay filtering. Note that SG filtering eliminates noise without compromising maximums and minimums.

Figure 3 shows differences among the three proposed filtering methods for the spectra obtained in the laboratory when filtering with a 10-point window in all cases. When processing the data, in the identification step, it is essential that filtering is the most suitable for the recognition method. In the percentile filtering (red) some noise remains, so the subsequent decomposition of the signal for identification will be more complex, increasing the processing time and increasing the error. The entire smoothing comparison was carried out with different orders in all the systems analyzed.

In FFT filtering, smoothing is very pronounced, and information may be lost and given the similarity in the response produced by some compounds, the probability of error increases since it reduces the effect of signal peaks and valleys.

Savitzky-Golay filtering is therefore the most suitable for this type of application. Not surprisingly, it is widely used in chemometric or mass spectrometric applications [1,13,18,19], where processes similar to those presented here are performed. In our case, this filter has been shown to be the most efficient to obtain the spectra because the main characteristics remain (i.e., significant peaks and valleys). For spectroscopic data, the method is effective to preserve both peak wavelength and width. The entire smoothing comparison was carried out with different orders in all the systems analyzed.

Performing a local polynomial regression around each point and creating a new one, smoothing the value for each data point results in a signal that is essentially more faithful than that obtained with the moving average window method. This approach tends to preserve the characteristics of the data such as the height of the peak as well as its width (which might be neglected if other methods are used instead). However, for large window sizes, the adjacent average of the resulting signal may differ too much from the input signal, while Savitzky-Golay may still retain the overall profile [16,20,21].

#### 2.1.4. Decomposition into Simple Functions

The use of statistical models to represent responses of experimental results [22,23] is very useful only when the parameters of the model are known beforehand. The most obvious advantage of using these parametric methods is the reduction in storage and processing time.

Goshtasby and O’Neill proposed in a 1994 paper [24] that, given a uniformly spaced sequence of individual data, it is possible to approximate the data to a sum of Gaussian functions with predetermined precision. Using this mathematical foundation in an accelerated computation process in the decomposition of the spectra obtained is the purpose of the procedure presented here.

To verify whether the fluorescence produced by different optical powers is such that it allows the identification of the compound based on the comparison with the spectrum generated under optimal conditions, the Gaussian or Lorentzian decomposition of the spectrum is a very useful technique because storing the data is relatively simple; it is enough to store the wavelength, width (function width when it acquires ½ of its peak value: FWHM—Half Width at Half Maximum) and multiplying factor (H) of each of the components. The combination of all the components will establish a result-function which will be compared against the measured signal.

In chemical analysis, it is assumed that the property of interest of a sample (be it concentration, viscosity, octane number, etc.) is related to the technique used to analyze that sample. In the case of detection and identification, the value provided by the technique (fluorescence) is compared with previously measured values. From this concept, a multivariable comparison model can be developed, to observe and compare properties of interest of multiple instrumental measurements. This technique allows quantification from non-selective measurements (that is, in the presence of common interferences in field measurement), and is especially useful in the analysis of spectrometric measurements.

In the case presented here, this method is useful both to quantify the concentration of the element that produces the fluorescence and to determine the fluorescence produced as a function of the incident optical power (extrapolated to the amount of pollutant), since it presents a linear behavior up to the nominal current.

Hydrocarbons are more or less long chains of carbon and hydrogens and the added elements depend on each manufacturer. This way the purpose of this decomposition is not to identify its constituent components or concentrations [25] but identify the spectrum showing by a measurement [15,18]. Performing a spectrum adjustment with Gaussians or Lorentzians [24,26] from a purely mathematical point of view, it is a very useful tool that can lead to valid identification.

To perform the curve fitting method successive iterations Levenberg-Marquardt [27,28] algorithm (LMA or just LM) is used to obtain a permissible error. As the number of Gaussians or Lorentzians increases, the result is optimized, but the amount of data to be stored increases and the identification process slows down. It is necessary to choose a figure-of-merit function that measures the agreement between the data and the model with a particular choice of parameters, to the avoid risk of overfitting [29], that is, the same model but with more explanatory variables. In the case presented here, it was preferable to choose the model with the least number of explanatory variables, since although the sum of squares of the residuals was always less with more variables, a slight increase in error was assumed to obtain a shorter calculation time.

The model parameters were adjusted to achieve a minimum in the merit function, producing the best fit parameters. The adjustment process was a minimization problem in many dimensions. The data were generally subject to measurement errors (called noise in the context of signal processing), so the typical data may not exactly fit the model being used, even when that model was correct. Therefore, we set an error less than the possible noise incorporated into the sample. Although there are several models with which to measure this error, the weighted sum of squares residuals (WSSR), or chi-square function has been used.

(1) $$\chi^{2}(a)=\sum_{i=1}^{N}{\left[\frac{y_{i}-y\left(x_{i};a\right)}{\sigma_{i}}\right]}^{2}=\sum_{i=1}^{N}\omega_{i}{\left[y_{i}-y\left(x_{i};a\right)\right]}^{2}$$

where the weights ω*_i_* are based on standard deviation σ*_i_*^2^. The result is shown below with a different number of components, until a small enough error is obtained, considering a maximum of six to validate the method, since, as previously stated, the aim was to achieve a system with the smallest number of data. As an example, in Table 1 the results of the decomposition of the diesel A spectrum when the nominal current flows through the LED is shown.

**Figure 4:**
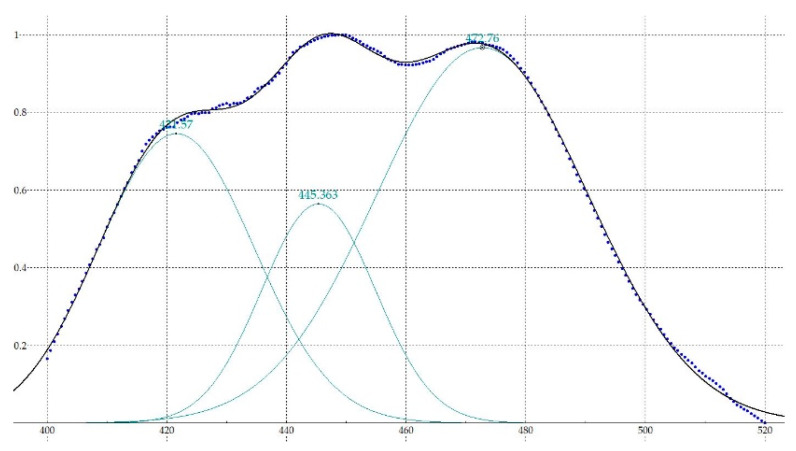
Spectrum after being bounded and normalized (dots), Gaussian functions (green) and sum of functions resultant (black).

Figure 4 shows the spectrum of diesel A after being bounded and normalized, the Gaussian functions and the result of the sum of these according to the error are indicated in Table 1.

In this way, it is possible to obtain the standard footprint of each of the compounds that want to be identified in the field analyzer. Each of these traces will have wavelengths (center in nm) and a number of components (Gaussian in the case presented) with their respective H and FWHM.

**Figure 5:**
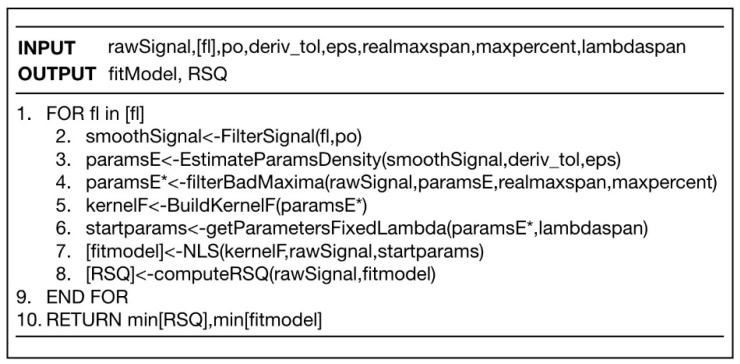
Pseudocode to implement decomposition of spectra into simple functions.

In order to check the validity of our decomposition method into simple functions method, we have designed the algorithm that we describe below (Figure 5).

The method we present is based on an educated grid-search to find the kernel function that best reproduces the observed signal. As a working hypothesis, we assumed that the raw signal was composed of the sum of a series of kernel functions plus a certain amount of noise. The method consisted of estimating where the maxima were in the most reliable way. We describe in detail below.

**1. First item. Initial Signal Filtering**

The first thing to do is to filter the function. Filtering requires two parameters: the order of the polynomial “po”" and the filter length “fl”. This is done with the function FilterSignal (fl, po) that implements the Sgolay algorithm. Sgolay filtering requires a polynomial order “po” to set a local polynomial over a length given by “fl”. Unfortunately, the result is very sensitive to parameter combinations. For example, a small fl gives a peaky curve, but if “fl” is too large it renders too smoothed curves and relevant information on possible local maxima is lost. By setting one of the parameters, e.g., the polynomial order to minimum po = 3, we can set the parameter “fl” free and obtain different smooth strengths.

**2. Potential Extrema Selection through Density-Based Clustering.**

Once the smoothed function “smoothSignal” is found for a given fl, we proceed to the estimation of local maxima by looking at the first and second numerical derivates. We searched points where the first derivative was approximately 0 with a given tolerance “deriv_tol”. The result was pairs of points {(lambda, h)} (position and height).

This technique works well for detecting maximums, minimums and chair points. The problem is that, depending on the level of smoothing, we can obtain false local extrema that would render false peaks.

Therefore, on this set of extreme candidates, we performed a density cluster analysis. The advantage of using a density-based method is that it allows us to detect when two maximum potentials are too close together. In this case, it is unlikely that these are real peaks. The DBSCAN [DBSCAN] algorithm uses two parameters: MinPts and eps. Here MinPts is the minimum number of points of a region to be considered dense while eps is the radius of the neighborhood around a point [DBSCAN]. This algorithm allows us to classify points in different clusters and in noise (those points not belonging to any cluster) according to their spatial proximity. 

In our case, we analyzed the proximity of maxima in the (lambda, h) space. We isolated cluster centroids and kept the point in case this was reported as noise. This allowed a fine-grain screening through a majority vote of possible extremes at a local level isolating the representative of the local community from extremes as a potential extrema.

**3. Screening of Local False Maxima.**

Once the positions and heights of the possible ends had been identified, the task was to discard any false maxima that may have been cast in the previous step. 

The idea was that if a large proportion “maxpercent” (e.g., 80%) of the points in a window of “span” around a maximum had lower h values, we discarded the points as a true maximum. This allowed us to skip false local maxima with very little computational cost.

**4. Building the Fitting Model.**

Once the maximums were set—let us say there were m of them—we used a representation of the signal function by means of a weighted sum of kernels:

(2) $$f(x,y)∼\sum_{i=1}^{m}g_{i}\left(x,y;\alpha \right).$$

If, for example, we used Gaussian kernels, we would have three parameters for each kernel: mean, variance and height (3m in total).

**5. Fitting by the NLS Method.**

The idea was to optimize these parameters with an educated guess for their initial and final values to direct the search in the most efficient manner. This guess was made in the following way. 

Estimation for lambda: lambda_o ± lambdaspan, being lambda_o the value we found in the previous stage.

On the other hand, both sigma and h were left free-oscillating between reasonable values (e.g., sigma = [0, max_x/2]) and h [0, max_y].

**Figure 6:**
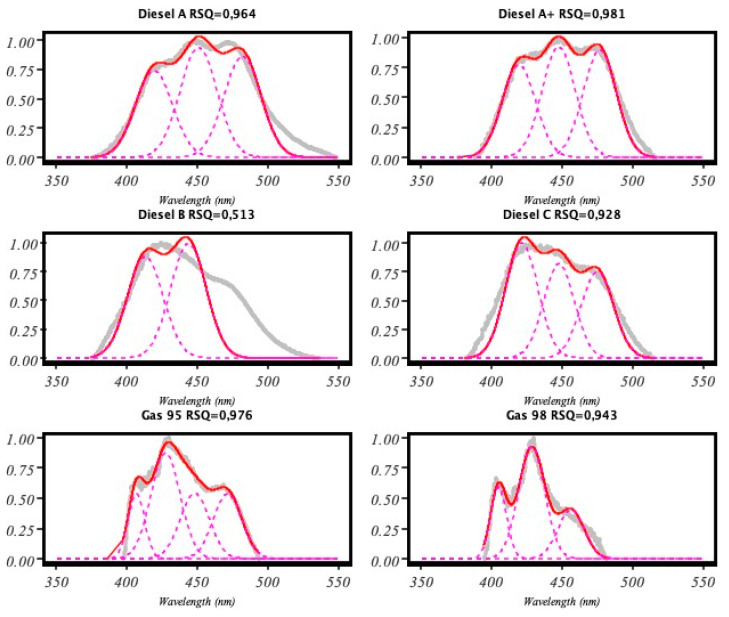
Results obtained from our implementation of the pseudocode shown in Figure 5.

As a result of the analysis, we got an R-squared (RSQ) value. Once we calculated the RSQ, we repeated the whole process again for different filtering values and found the best model. That was the one with the lowest RSQ (see Figure 6).

### 2.2. Identification Methods

This section describes the proposed method for the identification of the compounds stored in the database. Since the system is proposed for continuous detection, the processing should be as fast as possible, while it is possible to further optimize the result subsequently incorporating machine learning techniques.

**Figure 7:**
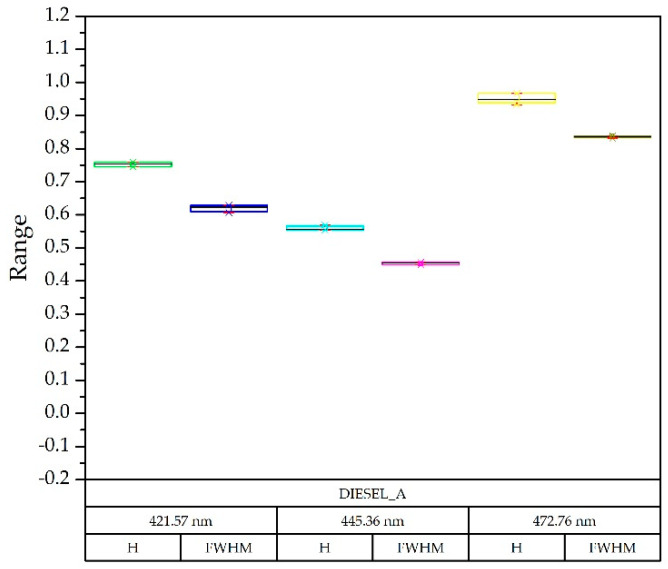
Box-plot diagram: comparison of the results obtained with the diesel A spectra with LED currents of 0.33, 0.44, 0.55 and 0.8 Amps, taking as reference the nominal current of 0.65 A. For each wavelength, the Gaussian in height (H) and width (FWHM—Half Width at Half Maximum) was identified.

Regarding the detection capacity for variable amounts of the pollutant, it has been previously shown that since the fluorescence produced is proportional to the amount of the compound to be detected [13], and also to the power of light emitted [1], this study used this second method so that less optical power was equivalent to a lesser amount of the compound [30]. Figure 7 shows the decomposition of the diesel spectrum A at different optical powers obtained from the injected current in a box plot diagram. The deviation from the nominal current was very small at all wavelengths.

Quantitative differences were significant, but when the spectra were normalized, the information of each of the compounds was preserved, providing robustness to the procedure. 

#### 2.2.1. Trial and Error System

**Figure 8:**
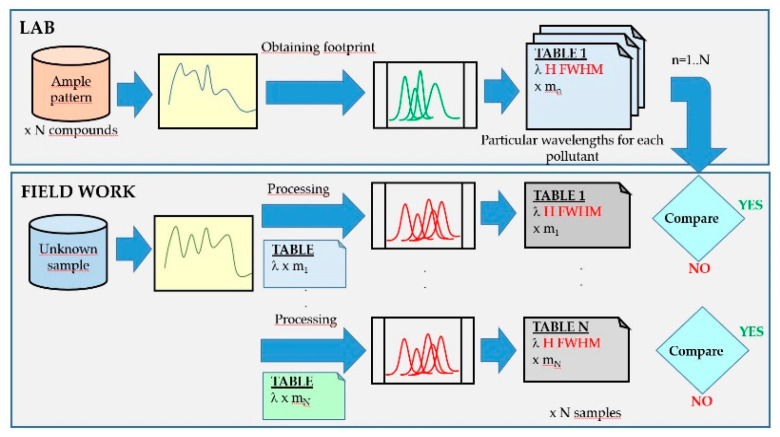
Detection scheme using fluorescence spectra by the trial and error comparison method: in the laboratory (upper part of the figure) and in the field analyzer (lower part of the figure).

This is a heuristic method widely used in the laboratory, since large databases and powerful computers are available, as well as enough time frames to carry out this task. The scheme of the logical identification system in which the spectrum obtained from a sample is compared with all in the database (Figure 8) and the generation of standard footprints is carried out in the laboratory. In the field analyzer, each spectrum of the sample taken is decomposed into Gaussian functions where the wavelengths have been fixed based on the pattern-footprint which it is intended to compare with. This process is repeated for all the element templates stored in the database. If the heights (H) and widths (FWHM) at those wavelengths match within an allowed error threshold, the program interprets the match. When there is coincidence with more than one compound, the one with the lowest error is taken.

**Figure 9:**
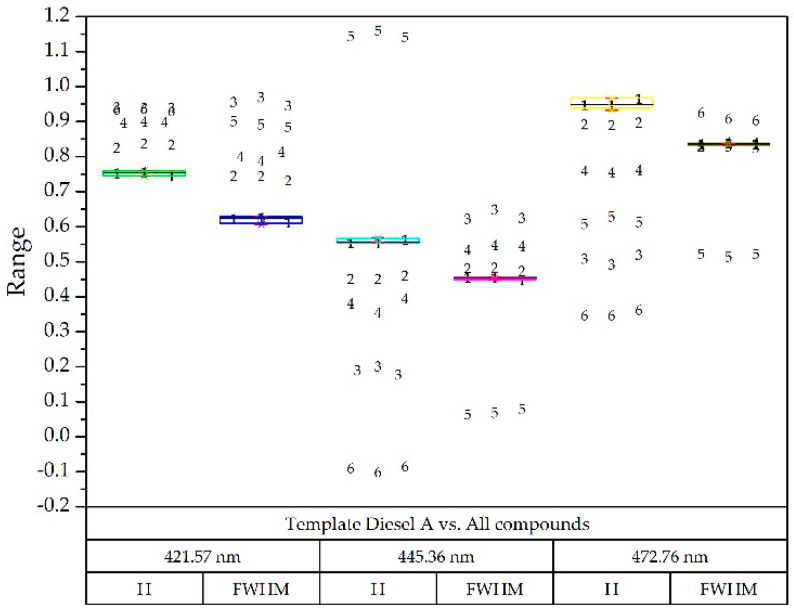
Box-plot diagram with reference diesel A (all of them at three optical powers): Diesel A (1), Diesel A with additives (2), Diesel B (3), Diesel C (4), Gasoline 95 (5) and Gasoline 98 (6).

To verify if the results were distinguishable enough from each other, the template of the diesel A compound (Figure 7) and the Gaussian decomposition of the rest of the compounds was superimposed in Figure 9 on a box-plot diagram, each of them with three optical powers. This example is illustrative since with only three Gaussians the problem is solved after having limited the wavelength range.

As shown in Figure 9, it was possible to implement a simple procedure for the identification of the sample compound, since the components were sufficiently separated in each wavelength, both in height (H) and width. (FWHM) [26,31].

#### 2.2.2. Proposed Method

Although the method we have presented is valid both in laboratory conditions and in the field as long as the number of compounds is not very high, it is necessary to develop another method that allows us to increase the database. This way it can detect multiple substances quickly, maximizing the computing capacity of current processors.

Once it has been verified that the decomposition into simple functions can be carried out quickly, the problem of comparing the results arrives from the fact that each compound to be compared presents a template at different wavelengths since it has different local maxima and minima.

**Figure 10:**
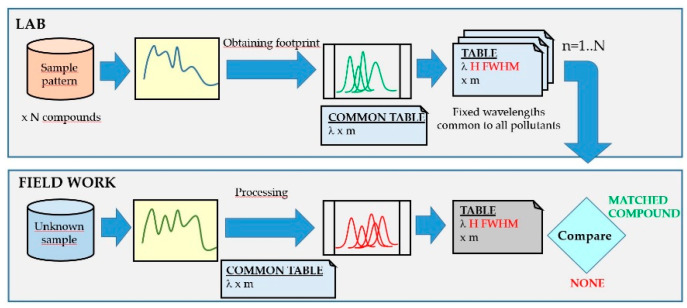
Detection scheme using fluorescence spectra by the proposed method: in the laboratory (upper part of the figure) and in the field analyzer (lower part of the figure).

The method presented here consists of performing a search on a common series of wavelengths to all the stored compounds since this would lead to a single processing of the spectrum, where the outputs obtained would be the heights (H) and widths (FWHM) obtained. The comparison procedure is then reduced to the subsequent numerical comparison, allowing multiple improvements and the introduction of learning processes (Figure 10).

**Figure 11:**
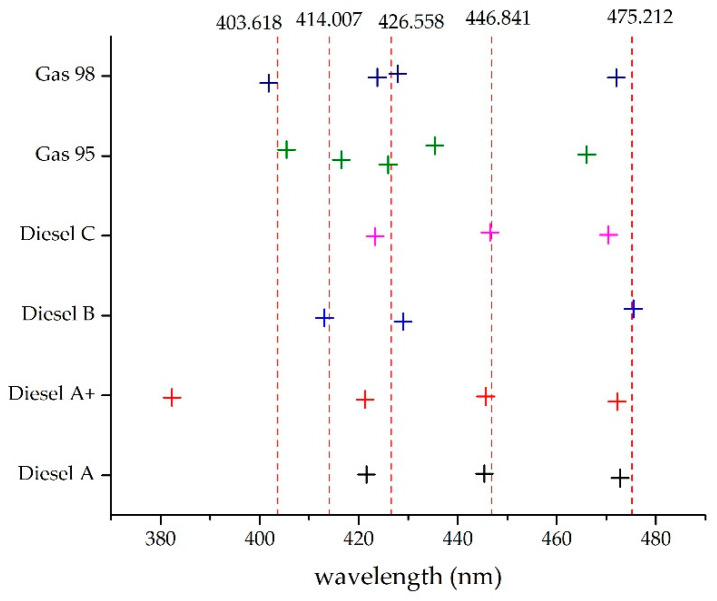
Determination of wavelengths to be used in the common template.

These common wavelengths could then be obtained from any computer implementation of the procedure; taking significant lengths as a starting point these were displaced in each iteration until an admitted error was achieved. If after a predetermined number of iterations a satisfactory result was not obtained, then an additional wavelength was added (Figure 11).

In view of the results, some of the components in certain compounds were greatly reduced, but it was more profitable in terms of computing time to have insignificant components than to add more functions.

## 3. Results

As previously stated, the wavelengths for a common template, constructed only with Gaussian functions, are shown in Table 2.

**Figure 12:**
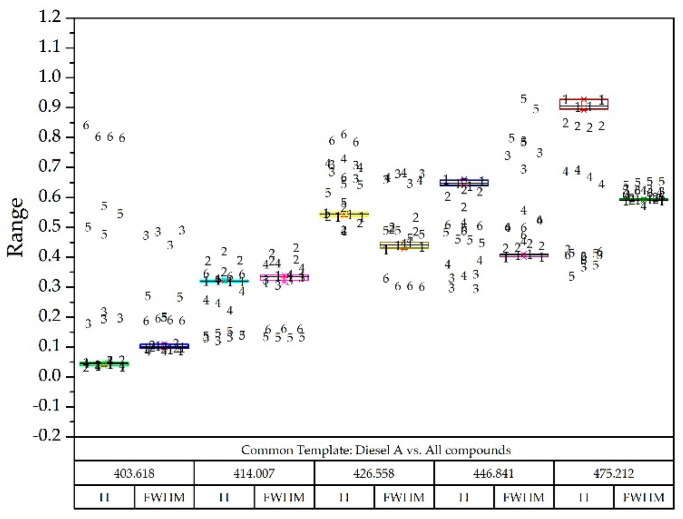
Diesel A box-plot diagram superimposed on all other compounds identified with numbers: Diesel A (1), Diesel A with additives (2), Diesel B (3), Diesel C (4), Gasoline 95 (5) and Gasoline 98 (6).

**Figure 13:**
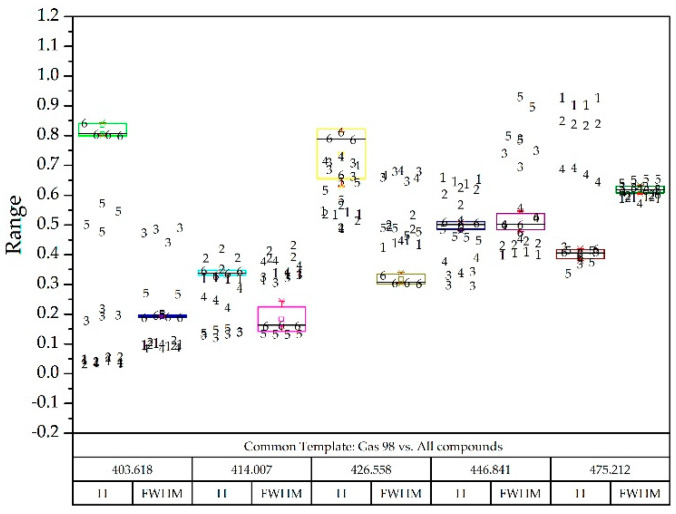
Gas 98 box-plot diagram superimposed on all other compounds identified with numbers: Diesel A (1), Diesel A with additives (2), Diesel B (3), Diesel C (4), Gasoline 95 (5) and Gasoline 98 (6).

Taking as reference the common template where all the compounds appeared and the normalized values they could take, and superimposing the box-plot diagram of the compound to be detected; two examples are seen in Figure 12 and Figure 13.

## 4. Conclusions

In view of the results obtained and based on the diagrams presented, it is possible to develop a CART decision tree from which an identification program can be obtained since there is enough difference in heights and widths of the functions in which each spectrum is decomposed.

Both the generation system of individual footprints of each compound and the field identification system can be carried out based on the concepts set forth herein, and the platform on which it is installed must be differentiated in each case.

Once the validity of the presented method has been verified, it is necessary to continue debugging it, so that greater precision and differentiation of each of the compounds can be obtained.

On the other hand, it is necessary to expand the library of stored spectra. Furthermore, the authors work on obtaining spectra of the contaminants not only depends on the amount of the compound, but also on its degradation. As they are volatile compounds, hydrogen-carbon chains pass into the air, and therefore the spectrum can present differences. This characteristic can also serve to determine the time that the pollutant has been exposed to air.

## Figures and Tables

**Table 1:** Adjustment results with Gaussians of the diesel A spectrum after five iterations.

	Diesel A		
Function	Wavelength (nm)	Height (H)	Width (FWHM)
Gaussian 1	421.57	0.744981	15.31856
Gaussian 2	445.36	0.563943	11.21524
Gaussian 3	472.76	0.966697	20.87015
χ^2^ = 0.0145169			

**Table 2:** Common template wavelengths.

Function	Wavelength (nm)
Gaussian 1	403.618
Gaussian 2	414.007
Gaussian 3	426.558
Gaussian 4	446.841
Gaussian 5	475.212
